# Recent Views Concerning the Treatment of Cancer, Based upon Its Nature1Remarks made at the 23d annual meetings of the Southern Surgical and Gynecological Association, held at Nashville, Tenn., December 13-15, 1910.

**Published:** 1911-04

**Authors:** Roswell Park

**Affiliations:** Buffalo, N. Y. 430 Delaware Avenue


### BUFFALO MEDICAL JOURNAL

VOL. LXVI. APRIL, 1911. No. 9

### ORIGINAL COMMUNICATIONS

### Recent Views Concerning the Treatment of Cancer, Based Upon Its Nature[1]

By ROSWELL PARK, M.D., LL.D.
Buffalo, N. Y.

IT is my hope that these remarks may be regarded as the continuation of those which I had the pleasure of making upon this same general topic at the last previous meeting. To plunge abruptly into the subject I would raise the following points for discussion. While all, or nearly all, of the present theories regarding the nature of cancer are unsatisfying there are, nevertheless, certain features which cannot be disregarded. Among these is the apparent lawlessness of cell action within these growths which, nevertheless, includes some features not generally appreciated. We must acknowledge, for instance:

(1) General variations in individual susceptibility or resistance.

(2) Quite comparable variations in some extrinsic agent, (such as we note in colon bacilli) or, more probably

(3) Both the above together.

Under No. 1 are included causes which act upon the various component cells of the part.

(4) Wide differences at different periods of life. Too frequently operations on young people are followed by speedy return or rapid dissemination, such as is rarely seen in elderly patients. Essential malignancy is constituted by something far more comprehensive and pervading than mere cell groupings and irregularities or histological resemblances. That which constitutes in some a rapidly growing cancer is in others, so far as appearances go, a much less significant affair; and we know not why.

The effects of heredity can be neither disregarded nor explained, but at best can only amount to the inheritance of some predisposition or favorable soil, or their prevalence in certain family strains, but inheritance has also to do with the so-called precancerous condition, and with so reducing tissue resistance that irritation and injury seem so often to be the direct and even the actual cause of cancer.

---

1. Remarks made at the 23d annual meeting of the Southern Surgical and Gynecological Association, held at Nashville, Tenn., December 13-15, 1910.

Despite our failure as yet to recognise and describe one or more distinct organisms it cannot be denied that the parasitic theory, in whole or in part, leaves less in the way of explanation still to be supplied than do any of the others. Nevertheless it will for some time to come prove almost as difficult to reconcile conflicting views and hypotheses as it is in religion or politics. The mental environment of the research worker seems to have almost as much to do with his mental processes and readiness to accept particular views as the physical state and environment of the cancer patient have to do with the course and progress of his cancerous lesion.

When an exceedingly competent pathologist and laboratory director can seriously state:

"That the hypothesis of the parasitic origin of tumors has had its day, because subject to direct experimental test it has been found incapable of proof, and inadequate to explain many facts disclosed in recent investigations" (*Wolbach, Report of Harvard Cancer Commission,* 1909, page 47) he must provoke a sad smile of disagreement among the many operating surgeons and clinicians, who come into daily contact with its many manifestations of infectiousness. He aptly illustrates, moreover, the differences of viewpoint between those who see and treat its active expressions among the living and those who see it only in the cadaver, or as prepared for histological study. He apparently ignores the heart sickening evidences among the former, and calmly, even wilfully disregards or sets aside all that has been done in experimental laboratories with cancer among lower animals.

Furthermore, while so iconoclastic with the parasitic theory he apparently forgets that it answers more of the mooted questions than any or all of the other theories, since assuming or granting all that has been written—or much of it—concerning the lawlessness of cell activities, their abnormal and changed characteristics, etc., as seen in cancer, not one of the other theories begins to explain satisfactorily why they act so lawlessly. Surely for such explanation some foreign, abnormal and extrinsic agency must be presupposed; it is an unavoidable and unnecessary fundamental concept in our work, and cannot be discarded. And inasmuch as both reason and analogy demand it why reject it in the case of cancer any more than we did in the cases of syphilis, sleeping sickness, or do yet with such diseases as scarlatina?

There is therefore no need to discard the belief that somewhere behind the mystery of cancer there lurks a living extrinsic agency—a *contagium vivum.* To hold to this working hypothesis will clarify rather than befog our vista.

With regard, then, to variations in susceptibility, certain patients evince a very low resisting power, and in them recurrence is an early and frequent event. That is in certain rare patients operation seems to do harm, by causing a dissemination of rapid and destructive type.

Thus I can never forget a case of a young mother, some thirty years of age, with a rapidly growing recent tumor in the breast. This I thoroughly extirpated, removing the underlying muscle and large area of skin, and cleaned out the axilla, and yet saw acute recurrence, in jacket form, almost before the wound was healed. Of this there had not been the slightest previous evidence.

There are two sets of factors at work in most cases, the external and the internal; as one predominates the other becomes less important. The more extrinsic the cause the more negligible becomes the other. Let us acknowledge, then, that an epithelial cell once become cancerous, assumes an identity and character of its own, and that it *ipso facto* ceases to be an ordinary epithelial cell, even if transplanted—as it is in every metastasis—it retains its acquired cancerous characteristics. Does any other theory explain any more lucidly why this is the case?

It is easier to reconcile than any other theory with a feature which is coming into more and more frequent consideration, namely, the so-called,

Precancerous Condition. Here it is not the disease but the predisposition or favorable soil which is inherited, at least apparently in certain cases. But what about those organs which are of latest embryological addition that are the most prone to succumb; *i. e.*, appendix, colon, intestinal diverticula?[1]

What, too, shall be said relative to infection from cancerous environment? In my remarks last year I called attention to the facility with which fresh rats developed sarcomatous thyroids after being confined in cages in which many months previously other rats with sarcoma of the thyroid had been kept, though meantime the cages had not been in use. What could be more significant, at least so far as this particular species is concerned? What, too, shall be said of the so-called cancer houses whose existence cannot be denied, though some deny the inference which naturally arises from their existence.

It is often stated that the physicians who come so constantly into contact with the disease never contract it, yet Budd saw in

---

1. In chronically inflamed and troublesome appendices, according to McCarthy, about one in two hundred are carcinomatous. In chronically hypertrophied prostates the proportion is much larger, while in large and rapidly growing goitres 3 to 4 per cent. are cancerous. Sutton found 10 per cent. of fibroid uterine tumors in women over fifty were undergoing some form of malignant degeneration. In 40 per cent. of cases of cancer of the fundus there are associated fibroid changes.

Originally innocent breast tumors notoriously create an environment favorable for cancerous development.

one small cancer hospital five of the medical staff die of cancer within a very few years. (*Löwenstein,* page 11.) Behla reports nineteen cases of apparent infection of previously healthy husbands by cancerous wives, and Gueillot reports twenty-three cases of cancer of the penis in husbands whose wives had uterine cancer. Cancer of the tongue has been reported after the use of so-called cancermilk. Guermonprez saw a cancerous wart develop on his own finger after being bitten during operation on a case of cancer of the face and mouth. Budd saw cancer develop on the tongue of a dog whose habit was to kiss and lick the lip of his master who had cancer of the lip.

What is the bearing of all the work recently done and now doing, with inoculations, toxines, vaccines, serums, and the like? Are they not all based on opinions that we have to do with some form or expression of infectiousness? In other words, do they not show that the men who are experimenting with their manufacture or are using them in treating the disease are demonstrating allegiance to the parasitic theory, whether they acknowledge it or not?

The following theories of tumor origin are among those now most under discussion and are mentioned simply in order to show what inseparable objections they create. There is, for instance, Waldeyer's theory of equilibrium between epithelial and connective tissue, the former being held in check by the latter, this check being relaxed by age? This is sheer fancy given a particular direction in the effort to align it with facts.

Cohnheim's theory of displacement of cells or cell groups from their normal relations during the process of development would call for the presence of displaced and adjacent cells throughout the body, while leaving yet unexplained the nature of the stimulus which calls them into activity as well as their possibilities when thus stimulated for unlimited growth. It must be said, however, that it would account for the relative frequency of such tumors as hypernephroma and retinal glioma in early childhood.

With some such hypotheses Ribbert combined a dislocation of cells, believing that epithelial cells artificially or accidently displaced and implanted in connective tissue might thrive and form tumors in their new locations. It is conceivable that epithelial cysts may be formed in this way, but no true tumors have ever been produced by any experiment with implantation or displacement. The Ribbert theory also includes a pre-supposition that some primary change or weakening occurs in the supporting connective tissue. Something of this kind may occasionally occur in embryonal rests; thus carcinoma of the appendix often appears in early life, the majority of cases before forty, the appendix being a rudimentary organ of comparatively recent origin.

There is also the Gametoid or Fusion theory that epithelial cells are penetrated by leucocytes and that by fusion of these elements a hybrid tissue is formed. Here the mitoses vary and the number of chromosomes is reduced, while their form is altered and assumes that of sex cells. The accompanying loss of chromatin is explained by Von Hansemann as an anaplasia, which only needs a stimulus or irritation to produce a tumor. Under this term, anaplasia, are included also a loss of power of differentiation and the acquisition of increased power of growth.

More and more of late another feature of comparative novelty has been introduced into the study of cancer—namely, its relation to diabetes. It has been known for some time that malignant tumors are especially rich in glycogen, and indeed their glycogen content has been regarded by some as an index of their malignancy. Recently Hirschfield has noted the relationship between diabetes and neoplasms of the female genitals, which often develop at about the time of inception of the constitutional condition, as if a diminished tolerance had an unfavorable effect upon carbohydrate metabolism; furthermore, it is known that extirpation of such tumors as those of the uterus and ovaries will often cause improvement in diabetic patients, sugar sometimes completely disappearing from the urine. Therefore according to Manders and others certain of these tumors may be regarded as diabetic equivalents.

*Rôle of irritation and trauma.*—The relation between these and cancer is as undeniable as it is yet irregular and variable. Cases showing this relationship I would divide as follows: into those which evince evidences of (*a*) intrinsic irritation. This must be regarded as including cases of so-called chronic inflammation as well as other forms of internal "reiz"; cancer of the breast following chronic mastitis; cancer of the pancreas, with or without gallstones or pancreatic calculi; cancer of stomach and intestine after ulceration; cancer of the tongue and mouth following leukoplasia and chronic and luetic lesions; psoriasis and various other skin lesions, including especially the keratoses and hyperkeratoses;—lupus carcinoma, which is a late lesion, about one out of every ten cases showing this tendency, which is more common in women than in men; cancer of the rectum, which often is the effect of malignant degeneration of previous hemorrhoids.

In connection with these forms of irritation certain other features more or less occasionally operative must not be forgotten, such as light upon the skin. It is worth while to remember that cattle fed on buckwheat become very sensitive to light rays, and develop skin disease when exposed to them. (Loeb.) The final stage of light action is the development of cancer in sensitised skins. It is thinkable that some substance is produced which sensitises the skin and provokes epithelial proliferation.

It is very doubtful whether there is anything peculiar to be ascribed to senility or to senescence of the epithelial cells; changes heretofore attributed to such conditions are more likely due to persistent and cumulative external agents—light, heat, irritation, etc., or their necrotising effects,—or else to certain stimulating effects on epithelium. Experience and experiment have shown that certain aromatic amido—organic derivatives may provoke an infiltrating growth of epithelium into underlying connective tissues, which is equivalent to cancer or to a precancerous condition (Loeb).

(*b*) *Extrinsic irritation.*—Of these there are so many forms that it is worth while to mention but a few illustrations; cancer of the tongue and mouth apparently induced by bad teeth; cancer of gall-bladder following the irritation of calculi; *x*-ray cancer following hyperkeratoses, cancer following burns, or developing on old superficial ulcers; aniline, paraffin, petroleum and arsenic cancers, some of these occurring in the bladder and being due to the elimination of aniline derivatives in the urine; Kangri cancer (seen in Kashmir) from use of the kangri or earthenware stove over the abdomen; betel-nut cancer in the cheek in women who chew the nut to excess; skin lesions, moles, wens, warts, etc., following continuous irritation, and taking the form more often of sarcoma; sailor's cancer, and that of chimney-sweeps; cancer following Bilharzia diseases, 50 per cent. of resulting tumors being malignant (Goebel), and due to stoppage of vessels by ova or the parasites whose continued presence leads to proliferative hypertrophy and imperfect repair. Besides these we see excellent illustrations in the horn—core cancer of cattle who are lashed to the plow by a rope around the horns, and furthermore, in trees and plants where may be seen the simplest forms of tumor or galls due to the presence of parasites. From a study of these xylomata very much may be learned.

Obviously and naturally some of the above mentioned factors really figure in both lists, especially so with the light rays, and of these particularly the short wave and ultra-violet, which produce senile keratoma, the Röntgen rays and even the rays of the sun.

What may be effected by a combination is shown in the effect which fluorescent stains are known to possess in sensitising living cells as well as certain ferments. The effect of irritating dusts may also be manifested externally as well as internally, this being equally true of coal tar products; while the gross effects of irritation produced by friction, or by heat are well known.

*Actual and serious traumatism.*—Of the effect of these in certain directions Dr. Coley has just given us a most excellent summary. The general subject has recently been treated in an important monograph by Löwenstein. (*Ueber Unfall und Krebskrankheit. Tübingen,* 1910. From Czerny's Heidelberg Institute für Krebsforschung.)

Let me quote a case which has recently come to my notice which may illustrate the rôle of trauma alone or of that and infection combined, which latter was easily permitted by the former.

A middle-aged nurse in New York City fell into an open coal hole in such a way that one of the corset steel stays in her corset was forced up into her breast, inflicting a penetrating wound. She had been previously well, and was of good family history. Almost before the wound was closed a cancer had rapidly developed in that breast, and in spite of its early and thorough removal local recurrence and metastasis had occurred, and when I heard of her a few months later she was dying of exhaustion, with scalp and body thickly studded with tumors which were breaking down, while there was every indication of a similar condition in the interior. In my own experience cases of this character are more likely to be sarcomatous than carcinomatous, and I think that the former is sometimes more rapid and the more to be dreaded.

Epithelium may change its type in consequence of constant irritation or of injury. Thus in the everted uterus columnar epithelium changes to a stratified squamous type, whose outer layers may even undergo a definite horny change. So, too, the epithelium of the conjunctiva and the nasal mucosa may change into a thicker and more stratified form, while horny and desquamated epithelium may collect in so-called cholesteatomata or pearly bodies. Such collections may also be found in the ureters, when calculi are present, and in the gall-bladder with or without calculi. In the respiratory passages papilloma and carcinoma arise in areas which have undergone such changes (*i. e.,* multiple reduplications of squamous epithelium.)

On the other hand, the change from squamous to columnar type is seen in the bladder and deep urethra, where papillary outgrowths are not uncommon, while when following chronic cystitis they are often associated with malignant degeneration.

All this has been described by Lubarsch (*Ueber heterotope Epithelwucherungen und Krebs., Verhandl. d. Deutsch. Path. Gesellschft.,* 1906,) under the name "heterotrophic proliferation," implying the occurrence of epithelium where it does not belong, *e. g.,* in the intestines between bundles of muscularis mucosa or quite beneath it. It is most frequent in the aged, and causes great difficulty of diagnosis between chronic inflammatory conditions and carcinoma. Diagnosis is made as much by history and gross appearances as by any method. In a large series collected by Lubarsch were fifty-two cases of stomach lesions, thirty-two in the intestines and thirty-six in the gall-bladder, all consisting of distinct proliferation of epithelium, with either hypertrophy or atrophy of the mucosa as a whole. In three out of five cases showing heterotrophic changes carcinoma was present. Out of six hundred and sixty-two cases of carcinoma of the stomach Haberfeld proved that at least one hundred and six, 16 per cent. developed upon a basis of ulcer.

He also proved that in primary carcinoma of the gall-bladder calculi were present in 70 per cent. of cases, while in secondary cancer they are very rare.

Again Lubarsch and others have shown that the injuries incidental to parturition have a positive influence in the subsequent development of squamous cell cancer of the cervix.

By virtue of the fact that the right primary bronchus makes nearly a straight line with the trachea the right lung is much more exposed to any infectious process. Out of one hundred and forty-six cases of cancer of the lung collected by Haberfeld ninety-four were primary on the right side.

Leaf's studies (*Zeitschft. f. Krebsforschung,* 1907), of breast cancer showed a history of abnormal lactation (excess or deficiency) in 71 per cent. of cases, and of injury in 32 per cent. while in 23 per cent. there was a family history of cancer.

At least forty cases of *x*-ray cancer are on record, mostly in young people. It notoriously follows the chronic dermatitis, telangiectasis and keratoses which occur only on exposed areas of the body. There is frequently a remarkable latent period, often several years. There is also a reversion to an earlier type where, while growth capacity is at a maximum, differentiation is at a minimum and the main changes occur in deeper tissues of the skin.

Similarity in forms of skin cancer, under various forms of injury, points to an identical kind of irritation. According to many they indicate that persistent irritations of the connective tissue which supports the epithelium are responsible for the acquisition by the latter of its malignant characteristics. Unfortunately these conditions cannot be duplicated experimentally, time is a most important element; in *x*-ray cancer perhaps ten years must elapse.

Certain recent discoveries have been made with regard to cancer in fish and a form of goitrous enlargement which often assumes sarcomatous form, and which is found to affect only fish in certain pools in various hatcheries, while those in other pools along side are exempt. When practically all the fish in one pool are infected we naturally look to their environment and its contents for explanation. Gaylord and Marsh have certainly determined this for individual hatcheries. They have found, moreover, that when fish thus affected are put into tanks, and in water containing so small a proportion of iodine or sublimate as to make one in three million solution the tumors cease to, or do not develop. Moreover, Gaylord took a litter of puppies to one of these infected pools, four of which drank the water, while two drank from an uncontaminated source; each of the four developed thyroidal enlargement, while the other two were exempt. From such facts as these you may draw your own inferences!

*Regarding the death rate from cancer.*—In spite of all effort to minimise the facts they show that the death rate per one hundred thousand is certainly increasing, while the total mortality is appalling. Thus in one year in Japan there were some twenty-five thousand deaths; in New York state the number was about seven thousand, while the total number of deaths during the same year in the United States was about forty thousand. As I have more than once remarked to our own legislators if seven thousand cattle died in our own state from a given disease there would be ordered a prompt investigation, and yet upon the loss of seven thousand human beings the legislature looks with apathy or with small interest.

Some interesting cancer statistics have recently come from Denmark (*Zeit. f. Krebsforsch.*, Vol. IX,) from which it would appear that in one hundred and two cases, *i. e.*, 10 per cent. of the total number, there was cancer among near relatives, or in the same family; a fact distinctly stated as not to be interpreted in favor of heredity, but rather as indicating in at least certain families a certain contagiousness. Four per cent. of cases evinced the same thing among persons closely associated, while 4 per cent. of cases also occurred in sisters. In sixty-five cases, *i. e.*, 7 per cent., of cancer of the alimentary tract one half of them had near relatives with the same form of cancer. Or including abdomen and liver these figures might be placed at fifty-four out of seventy-six persons, *i. e.*, 70 per cent. of those thus affected.

The German Cancer Commission, too, have taken the ground that since in about 14 per cent. of a large total of cases the parents suffered from a similar disease this should be regarded as of infectious character. In man we certainly note occasionally peculiar family predisposition to tumors of various kinds; thus in one family I have known of sixteen instances in two generations. This same thing is noted in small animals.

### TREATMENT.

To what extent have we advanced in the therapy of malignant disease? This is a most important question, especially in view of methods of investigation or inoculation which have recently come into vogue.

It was in the Buffalo Laboratory that it was first discovered that the serum of animals that had undergone spontaneous retrogression of inoculation cancer, as a small percentage did, would cause decrease in or disappearance of similar growths in other animals of the same species. It was upon these facts here constituted by Gaylord and Clowes that Hodenpyl based his first announcement of success in treating cancer by injection of serum taken from a human case of spontaneous subsidence of this disease. How luring and yet how disappointing this announcement has proven is a matter of very recent history which must be still fresh in your minds. While it probably contains the germ of a great truth much time and study must yet be devoted to elucidating the mystery which it contains. In the Buffalo Laboratory, as in others, encouraging results have been obtained by injection of enzymes, in both fluid and solid state, which have been prepared from the tissues of malignant tumors, but all this opotherapy is yet so completely in the experimental stage that one would be wrong in drawing conclusions, or in doing more than treating selected patients during the experimental period.

Of all other internal remedies or drugs and non-surgical procedures I have, with others, for many years regarded *arsenic* as that substance which has more nearly a selective or elective action on the cancer cell than any other. That others share this belief is shown by the prominence which has of late been given in various parts of the world to what I may call, arsenotherapy; in all of which I see only a confirmation of views expressed by myself at least twenty years ago. As yet, however, the best that can be said of this is that it is of value in certain cases, and one should never hesitate to combine with it any form of surgical treatment, and to follow the latter with arsenical preparations and perhaps with *x*-ray treatment.

To operative measures we have yet to turn as offering by far the most hopeful expedient in recent cases, and perhaps the only possible one in the late cases. While we all well know that the laity themselves are most to blame in declining to accept these measures we must yet realise that it is our paramount duty to show them that *danger lurks in delay and time lost, rather than in the operation itself.*

Excision, then, is the ideal method in theory, if only it can be made radical and thorough, even though necessarily much healthy tissue be sacrificed, in which respect it is perhaps crude; possibly in some cases tissues are sacrificed that might furnish some sort of resistance as against inroads of the disease, while lymph spaces are certainly opened up and fresh infection possibly thus invited, assuming, of course, that there is about the disease an infectious element. (In this respect I would quote myself as stating that for me every metastasis has the force and significance of a fresh inoculation made under favorable circumstances.) Thus Meller, discussing operations for cancer about the face and lips, shows that local recurrence in and about the scar is much more common and takes place earlier than in the lymph nodes, which means that we often fail to remove all the affected tissues, and that incomplete operation may even do more harm than good? This he concludes is the result when the knife is wielded by an unpractised, untrained surgeon (*Zeit. f. Krebsforschung,* 1907, page 64). All of which goes to show that the technic of operations for cancer must be based on the most accurate knowledge of its characteristics; also, as a corollary that all operations which cannot be made complete and thorough should be made with the cautery knife.

And, here, now I may call your attention to one of the strangest inconsistencies in this whole question and controversy—namely, that many of the men who adhere most strictly to these practices and to this improved technic, as well as some of those who are playing with serum, or are inoculating various cancer products, still affect to disregard the parasitic hypothesis while yet basing their every precaution upon its truth.

What shall be said of other methods of treatment? There remain to be mentioned the use of *caustics,* the employment of *cathode and radium emanations and fulguration. Caustics find* their principal recommendation in that their resulting reaction serves apparently to block lymph channels, and by thus causing violent and extensive reaction, produce a sanitary cordon of fresh inflammatory tissue which walls off the area of pathologic activity and may also destroy scattered cancer cells. This statement puts them in their best possible light; obviously they are only available on affected surfaces, but the above features are so conspicuously of theoretical advantage that escharotics are beginning to commend themselves again in the hands of educated and trained surgeons. Heretofore they have been too exclusively in the hands of charlatans and quacks who, themselves afraid to operate, have used these substances upon patients who were afraid to undergo operation. Among the escharotics should be included first of all the use of the cautery knife in cases where a clean excision by careful dissection may not seem practicable.

Among them also should be included *liquid air* and *carbon dioxide snow,* the former almost impracticable, and the latter serving admirably in various skin lesions including, conspicuously, lupus erythematosus, and ranging from these to rodent ulcer and small epitheliomata, including also many nævi and other skin lesions. (See *Pisko, N. Y. Med. Jour.*, Feb. 4, 1911, page 215.)

*Fulguration* depends for its efficacy not on its actinic or light effects, but on a rude and unreliable cell destruction, which if effective is difficult, elaborate and painful, and which is usually impracticable because of the expensive apparatus required and dissatisfaction as concerns end results.

*Cathode rays* and *x-rays* can be made absolutely destructive to superficial growths if used to excess, but the *x*-ray is in effect a two-edged sword; nevertheless it may be used as the principal agent, as a preliminary therapeutic measure preceding operations, or as a post-operative protection. The first and the third of these uses are, in trained hands, often very successful; the second is a disappointing and even a dangerous measure. Again, cathode rays may be used for their destructive and specific effects for relief of pain, without reference to the former, and to aid prompt absorption of exudate or scar tissue. I have convinced myself that they are of great value after operations where portions of the abdominal viscera or of abdominal growths have been removed on account of cancer. Here they seem, at least, to retard recurrence and I repeat, in my own experience, to apparently prevent it. They have, moreover, a special field of usefulness in certain locations as about the face, the vulva, etc., they should never be used by those who are not as keenly alive to their dangers as to the benefits resulting from such use. To these dangers which are so well known to you I will not now make further reference.

*Radium.* On account of its extreme cost radium therapy is not likely to become popular in the near future. In the hands of a very few men, like Abbe, it seems to have accomplished much in a few cases. I have obtained benefit and apparent cure from its use in a very few cases of superficial cancer and lupus. Its use is not unattended by risk, and needs to be controlled as does that of the cathode rays.

After all, discussion of the therapy of malignant disease is too much like trying to entertain you with a meditation upon death. There is as yet little satisfaction to be gained by any but the most radical surgical procedure, and this to be completely successful should be early. Finally, I am tempted to try to lift from the profession the opprobrium of failure to recognise cancer, especially in its internal forms, at an early enough period to make operation generally successful, by reminding you of that to which I called your attention a year ago, and to which the profession are all too blind—namely, that *cancer as a disease has absolutely no symptomatology of its own.* *Signs* it has by which it may be easily recognised when superficial, but when within the body cavities it is made evident to sight or touch by unmistakable signs it is then indeed late to expect a cure from any source.

430 DELAWARE AVENUE.

